# A Comparative Field Evaluation of the Effect of Growth Performance Between Porcine Circovirus Type 2a (PCV2a)- and PCV2b-Based Bivalent Vaccines Containing PCV2 and *Mycoplasma hyopneumoniae*

**DOI:** 10.3389/fvets.2022.859344

**Published:** 2022-06-24

**Authors:** Hyejean Cho, Taehwan Oh, Jeongmin Suh, Chanhee Chae

**Affiliations:** Department of Veterinary Pathology, College of Veterinary Medicine, Seoul National University, Seoul, South Korea

**Keywords:** *Mycoplasma hyopneumoniae*, porcine circovirus type 2, porcine circovirus type 2a (PCV-2a), porcine circovirus type 2b (PCV-2b), bivalent vaccine

## Abstract

The objective of this study was to compare two different bivalent vaccines containing porcine circovirus type 2 (PCV2) and *Mycoplasma hyopneumoniae*. One vaccine contained PCV2a and the other contained PCV2b, and both were administered on a farm suffering from subclinical PCV2d infection and enzootic pneumonia. A total of 180 pigs were randomly divided into 3 groups (60 pigs per group; male pigs = 30 and female pigs = 30). Bivalent vaccination significantly improved growth performance in both vaccinated groups as compared to the unvaccinated (UnVac) group. Growth performance measured by body weight and average daily weight gain (ADWG) was not significantly different between the two bivalent-vaccinated groups (VacA and VacB). Both bivalent vaccines elicited high levels of neutralizing antibodies and interferon-γ secreting cells (IFN-γ-SC) against PCV2d, leading to a reduction in the levels of PCV2d blood viral load as compared to unvaccinated animals. Similarly, both bivalent vaccines elicited high levels of IFN-γ-SC against *M. hyopneumoniae* that reduced the level of *M. hyopneumoniae* laryngeal viral loads as compared to unvaccinated animals. Significant differences in severity of lung and lymphoid lesions were observed in both vaccinated groups as compared to the UnVac group. These comparative field data demonstrated that both bivalent vaccines are good candidates for controlling subclinical PCV2d infection and enzootic pneumonia in swine farms suffering from an existing infection.

## Introduction

Porcine circovirus type 2 (PCV2) is the main etiological agent of porcine circovirus-associated disease (PCVAD) (1, 2). Since the introduction of PCV2 vaccines to the market, the clinical form of PCVAD has dramatically decreased, but subclinical PCV2 infection remains the most identified problem in the field (3). This subclinical PCV2 infection is measured and observed through only one clinical sign; growth retardation (4, 5). Meanwhile, *Mycoplasma hyopneumoniae* is recognized as the primary causative agent of the so-called enzootic pneumonia. The disease causes significant economic loss, also due to growth retardation and the increased cost of antimicrobial medication (6, 7).

Complications from both subclinical PCV2 infection and enzootic pneumonia were involved in the porcine respiratory disease complex (PRDC). PRDC devastates swine herds through decreased growth rate and poor feed efficiency results in an extended time to market (8, 9). Vaccination with a combination product is a great option in the simultaneous control of these two pathogens as it reduces both labor involved and animal stress during vaccination. Therefore, a single-dose-combined vaccine of PCV2 and *M. hyopneumoniae* is a better choice in the control of PRDC in herds suffering from severe respiratory disease.

A homologous vaccination and challenge (matched genotype) may offer better protection than a heterologous (non-matched genotype) vaccination and challenge for PCV2 (10). However, there is strong evidence of a higher immunogenicity of PCV2a when compared with PCV2b. Therefore, it is misleading to consider vaccine performance only in terms of similarity between field and vaccine PCV2 strains (11, 12). In global field situations, PCV2d has become the predominant genotype in pig populations over both PCV2a and PCV2b (13–16). A new genotype in the field may be more pathogenic, but it is likely to be properly controlled by the present vaccines if properly adopted and in the context of good farming practices (17). Nevertheless, only PCV2a- and PCV2b-based bivalent vaccines also containing *M. hyopneumoniae* are commercially available (18, 19). For this reason, PCV2b-based bivalent vaccines are of particular interest for swine producers and practitioners as PCV2b is genetically closely related to PCV2d (formerly called “mutant PCV2b”) (20). To date, a comparison between PCV2a- and PCV2b-based bivalent vaccines that also contain *M. hyopneumoniae* has not been conducted under field conditions. The objective of this study, therefore, was to compare PCV2a- and PCV2b-based bivalent vaccines containing PCV2 and *M. hyopneumoniae* for each of these clinical, immunological, microbiological, and pathological outcomes in a farm suffering from subclinical PCV2d infection and enzootic pneumonia.

## Materials and Methods

### Ethical Statement

All of the methods were approved by the Seoul National University Institutional Animal Care and Use and Ethics Committee (SNU-210518-3).

### Farm History

The clinical field trial was performed on an 800-sow, farrow-to-finish swine farm that carries out an all-in-all-out production system. A stable status of the porcine reproductive and respiratory syndrome (PRRS) was maintained with the absence of active porcine reproductive and respiratory syndrome virus (PRRSV) circulation (only multiple parity sows were seropositive animals). Sows were not previously immunized against PCV2 and *M. hyopneumoniae*. The farm was selected on the basis of subclinical PCV2 infection and enzootic pneumonia. The farm consistently had respiratory problems due to poor growth in the late post-weaning and growing stages. Clinical signs first appeared at approximately 8–11 weeks of age and reached peak mortality (~3–5%) between 10 and 15 weeks of age. PCV2d was detected in serum from 3 pigs with poor growth, where log_10_ DNA copies/ml ranged from 2.46 to 3.34. These values were consistent with the definition of subclinical PCV2 infection (21, 22). Through pre-trial investigations, a PCV2 serological profile was identified that presented an increase in antibody titers starting around 8 weeks of age; 7–16-week-old pigs with poor growth were also PCV2 PCR-positive in tested blood samples. *M. hyopneumoniae* serology was positive in 8–16-week-old pigs with respiratory signs. Moreover, the laryngeal swabs of the same pigs were PCR-positive for *M. hyopneumoniae* on the farm. Histologically, mycoplasmal lung lesions characterized by peribronchiolar and peribronchial lymphoid tissue hyperplasia were observed in 4 out of 5 submitted pigs. Lymphoid depletion without granulomatous inflammation was also observed in 3 out of 5 submitted pigs. Pre-trial diagnostic results indicated subclinical PCV2 infection and enzootic pneumonia.

### Experimental Design

To reduce sow variation, six pigs (21-day-old) per sow were randomly selected by the random number generator function (Excel, Microsoft Corporation, Redmond, WA, USA) and evenly allocated to each of the three groups. A total of 180 pigs were randomly divided into 3 groups (60 pigs per group; male pigs = 30 and female pigs = 30) within the same software and function (Table 1).

**Table 1:** Field experimental design.

**Groups**	**No. of pigs**	**Vaccine**	**Dosage**	**Age (days)**
VacA	60	Porcilis^®^ PCV M Hyo	One (2.0 mL)	21
VacB	60	Circo/MycoGard^®^	One (1.0 mL)	21
UnVac	60	Phosphate buffered saline	One (2.0 mL)	21

At 0 day post-vaccination (dpv, 21 days of age), pigs in the VacA group were intramuscularly vaccinated with a 2.0 ml dose of the bivalent vaccine containing PCV2a and *M. hyopneumoniae* (Porcilis^®^ PCV M Hyo, lot no. A114A01, expiration date: 23 June 2022, MSD Animal Health, Boxmeer, Netherlands) at the right side of the neck in accordance with the manufacturer's directions. According to the manufacturer's instruction, pigs in the VacB group were intramuscularly vaccinated with a 1.0 ml dose of the bivalent vaccine containing PCV2b and *M. hyopneumoniae* (Circo/MycoGard^®^, serial no: CMG-21006, expiration date: 20 January 2023, Pharmgate Animal Health, Wilmington, NC, USA) in the same anatomical location. Pigs in the unvaccinated (UnVac) group were injected with 2.0 ml of phosphate-buffered saline (PBS, 0.01 M, pH 7.4) in the same anatomical location. Sample collection of blood and laryngeal swabs were performed at 0 (21 days old), 28 (49 days old), 49 (70 days old), and 91 (112 days old) dpv.

### Clinical Observations

The pigs were daily monitored for abnormal clinical signs and weekly scored using scores ranging from 0 (normal) to 6 (severe dyspnea and abdominal breathing) (23). The mortality rate was calculated as the number of dead pigs by the number of pigs initially assigned to the group within the batch. Necropsy was performed on dead or culled pigs throughout the study. Including palpation, injection site reaction was evaluated 24 h post-vaccination. Observers were blinded to the type of vaccine status and vaccination.

### Average Daily Weight Gain

The live weight of each pig was measured at 21 (0 dpv), 70 (49 dpv), and 175 (154 dpv) days of age. Over two time periods, (i) between 21 and 70 days old and (ii) between 70 and 175 days old, the ADWG (grams/pig/day) was analyzed. During the different production stages, ADWG was calculated as the difference between the starting and final weight divided by the duration of the stage. Data for culled or dead pigs were also included in the calculation.

### Quantification of PCV2d DNA in Blood

For DNA extraction from collected serum samples, the commercial kit was used (QIAamp DNA Mini Kit, QIAGEN). An extracted DNA sample was used for the quantification of PCV2d genomic DNA copy numbers by real-time PCR (24). The forward and reverse primers (5′-GTA TTC AAA GGG CAC AGT GAG G-3′ and 5′-GCA CCA TCG GTT ATA CTG TCA AGA AA-3′) and probe specific for PCV2d (5′-FAM^TM^-CAT CAT GTC CAC ATT CCA G-3′ Black Hole Quencher) were designed for detecting specific Capsid-coding region of PCV2d only (24).

### Quantification of *M. hyopneumoniae* DNA in the Larynx

For DNA extraction from collected laryngeal swabs, the commercial kit was used (QIAamp DNA Mini Kit, QIAGEN). An extracted DNA sample was used for the quantification of *M. hyopneumoniae* genomic DNA copy numbers by real-time PCR (25). The forward and reverse primers (5′-TTG ACT GCT ATC TTT GCA CGA TAA G-3′ and 5′- ACA ATA ATT GCT GAC CGT GGC-3′) and probe (5′-FAM-TGT CCA CTG CTG CAA ATA TTC GAT TTC TTG AA-TAMRA-3′) were used to detect *M. hyopneumoniae* (25).

### Serology

Enzyme-linked immunosorbent assay (ELISA) was performed to measure antibodies against *M. hyopneumoniae* (M. hyo. Ab test, IDEXX Laboratories Inc.) and PCV2 (Ingezim CIRCO IgG, Ingenasa, Madrid, Spain). According to the instruction of the manufacturer for each kit, serum samples were recorded as positive for *M. hyopneumoniae* antibody if the S/P ratio (sample-to-positive ratio) was ≥0.4. For anti-PCV2 antibodies, samples were recorded as positive if the reciprocal ELISA titer was >350. A serum virus neutralization test against PCV2d was used to test the serum sample (26–28).

### Enzyme-Linked Immunospot Assay

The number of PCV2d-specific and *M. hyopneumoniae*-specific interferon-γ secreting cells (IFN-γ-SC) in peripheral blood mononuclear cells (PBMCs) was measured by ELISPOT assay (24, 29). Briefly, 100 ml containing 2 × 10^6^ PBMCs in Gibco Roswell Park Memorial Institute (RPMI) 1640 medium supplemented with 10% fetal bovine serum (HyClone Laboratories, Inc., SelectScience, Bath, UK) were seeded onto plates and precoated overnight with anti-porcine IFN-γ monoclonal antibody (5 μg/ml, Mabtech, Mariemont, OH, USA) and incubated with PCV2d (20 mg/ml), *M. hyopneumopniaee* (4 mg/ml), phytohemagglutinin (10 mg/ml, Roche Diagnostics GmbH, Mannheim, Germany) as a positive control, or PBS as a negative control for 20 h at 37°C in a 5% humidified CO_2_ atmosphere. The wells were washed five times with PBS (200 ml per well) and thereafter, the procedure followed instructions of the manufacturer using a commercial ELISpot assay kit (Mabtech). The spots on the membranes were read by an automated ELISpot reader (AID ELISpot Reader, AID GmbH, Strassberg, Germany). The results were expressed as the number of responding cells/million PBMC.

### Pathology

To estimate the percentage of the lung affected by pneumonia, two pathologists (Chae and one graduate student) at the Seoul National University (Seoul, Republic of Korea) scored the severity of macroscopic lung lesions as previously described (23). The collected lung and lymphoid tissue sections were examined and scored by two blinded veterinary pathologists; the severity of peribronchiolar and perivascular lymphoid tissue hyperplasia by mycoplasmal pneumonia lesions, on a scale of 0–6 (30). Based on lymphoid depletion and granulomatous inflammation, lymphoid lesion severity was also scored on a scale of 0–5 (31).

### Statistical Analysis

Real-time PCR and neutralizing antibody data were recalculated to log_10_ and log_2_ values, prior to statistical analysis. The collected data were assessed by the Shapiro-Wilk test for a normal distribution. Then, one-way ANOVA was performed to determine if there were statistically significant differences between different groups at each time point. For further evaluation, a *post-hoc* test for a pairwise comparison with Tukey's adjustment was conducted with a statistical significance result from a one-way ANOVA test. In case of the normality, assumption was not met and the Kruskal-Wallis test was performed. Results, which showed a statistical significance from the Kruskal-Wallis test, were further evaluated with the Mann-Whitney test to include Tukey's adjustment to compare the differences among the groups. The value of *p* < 0.05 was considered significant and reported in *p*-values.

## Results

### Clinical Signs

Two vaccinated animals from the VacA and VacB groups had significantly (*p* < 0.05) lower respiratory signs than those of unvaccinated animals from the UnVacA group at 14–112 dpv. There were no differences in respiratory signs between the two vaccinated groups (VacA and VacB).

### Average Daily Weight Gain

No difference in mean body weight was observed between the vaccinated and unvaccinated animals at the time of vaccination. During the growing period (70–175 days of age), the ADWG of the vaccinated groups (VacA and VacB) was significantly (*p* < 0.05) higher than that of the unvaccinated group (UnVac). Overall (3–175 days of age), the ADWG of the vaccinated groups (VacA and VacB) was significantly (*p* < 0.05) higher than that of the unvaccinated group (UnVac) (Table 1).

### Mortality

A total of 2 pigs died in the VacA group of severe pneumonia as determined by a combination of *M. hyopneumoniae* and PCV2d that was detected with PCR testing, and *Pasteurella multocida* was isolated from the lungs at 77 and 85 days of age. One pig from the VacB group died of pleuropneumonia caused by coinfection with *Actinobacillus pleuropneumoniae* and *Glaesserella parasuis* that was determined through isolation from the lungs at 81 days of age. A total of 4 pigs were died in the UnVac group; two pigs were died of bronchopneumonia as determined by a combination of *M. hyopneumoniae* that was detected with PCR, and *P. multocida* and *Trueperella pyogenes* that were isolated from the lungs at 69 and 85 days of age. The other two pigs were died from bronchopneumonia as determined by a combination of *M. hyopneumoniae* and PCV2d that were detected with PCR, and *G. parasuis* that was isolated from the lungs at 81 and 86 days of age.

### Quantification of PCV2 in Blood

**Figure 1:**
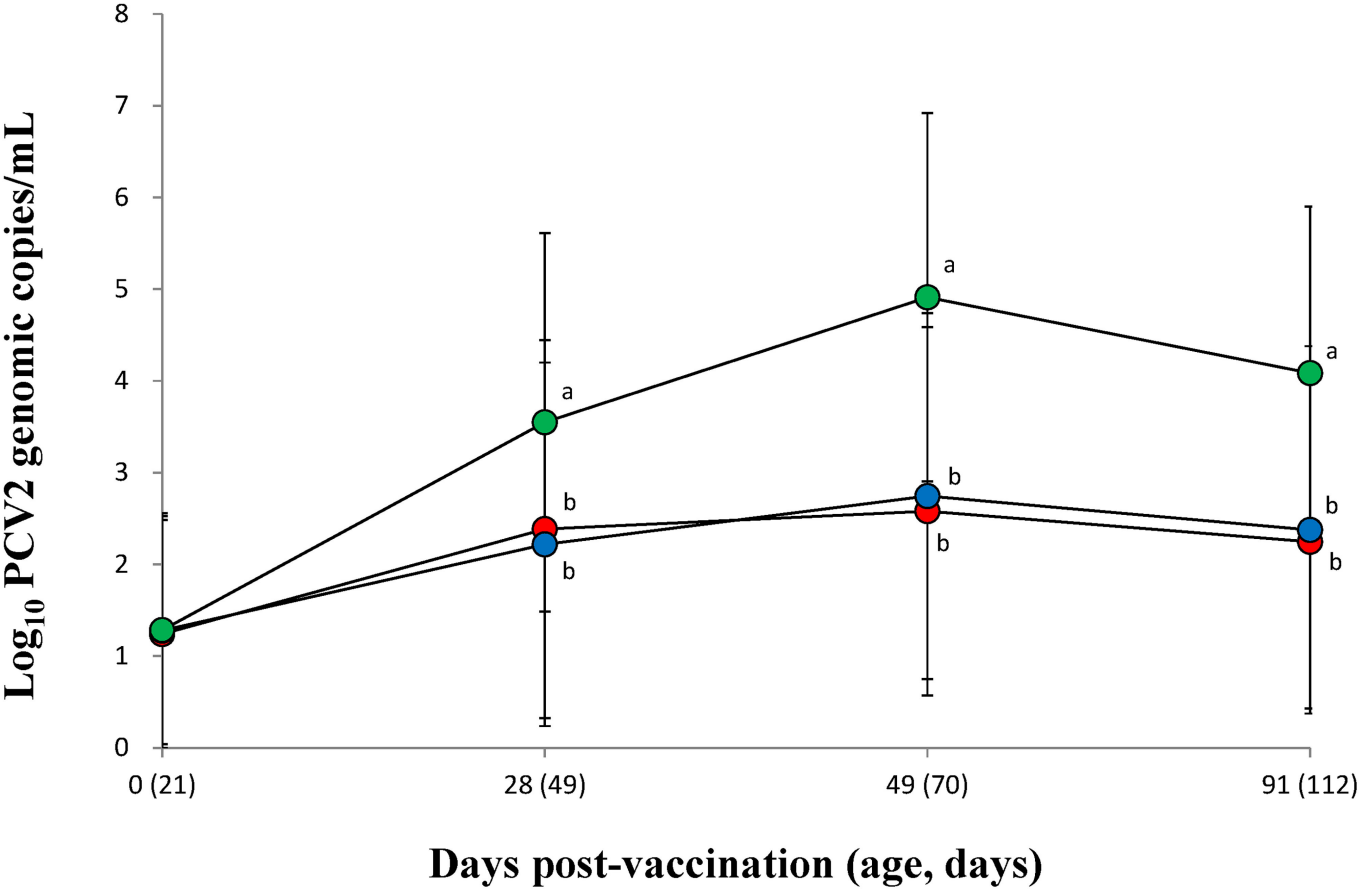
Mean values of the genomic copy number of PCV2d DNA in serum of pigs from the VacA (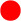), VacB (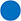), and UnVac (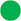) groups. Variation is expressed as the standard deviation (SD). Different superscripts (a and b) indicate a significant (*p* < 0.05) difference between vaccinated (VacA and VacB) and unvaccinated (UnVac) groups.

The PCV2 DNA blood loads from vaccinated groups (VacA and VacB) were significantly (*p* < 0.05) lower than that of unvaccinated groups (UnVac) at 28, 49, and 91 dpv (Figure 1). Two of the vaccinated groups (VacA and VacB) had comparable PCV2 DNA loads in their blood throughout the entire field trial.

### Quantification of *M. hyopneumoniae* DNA in Larynx

**Figure 2:**
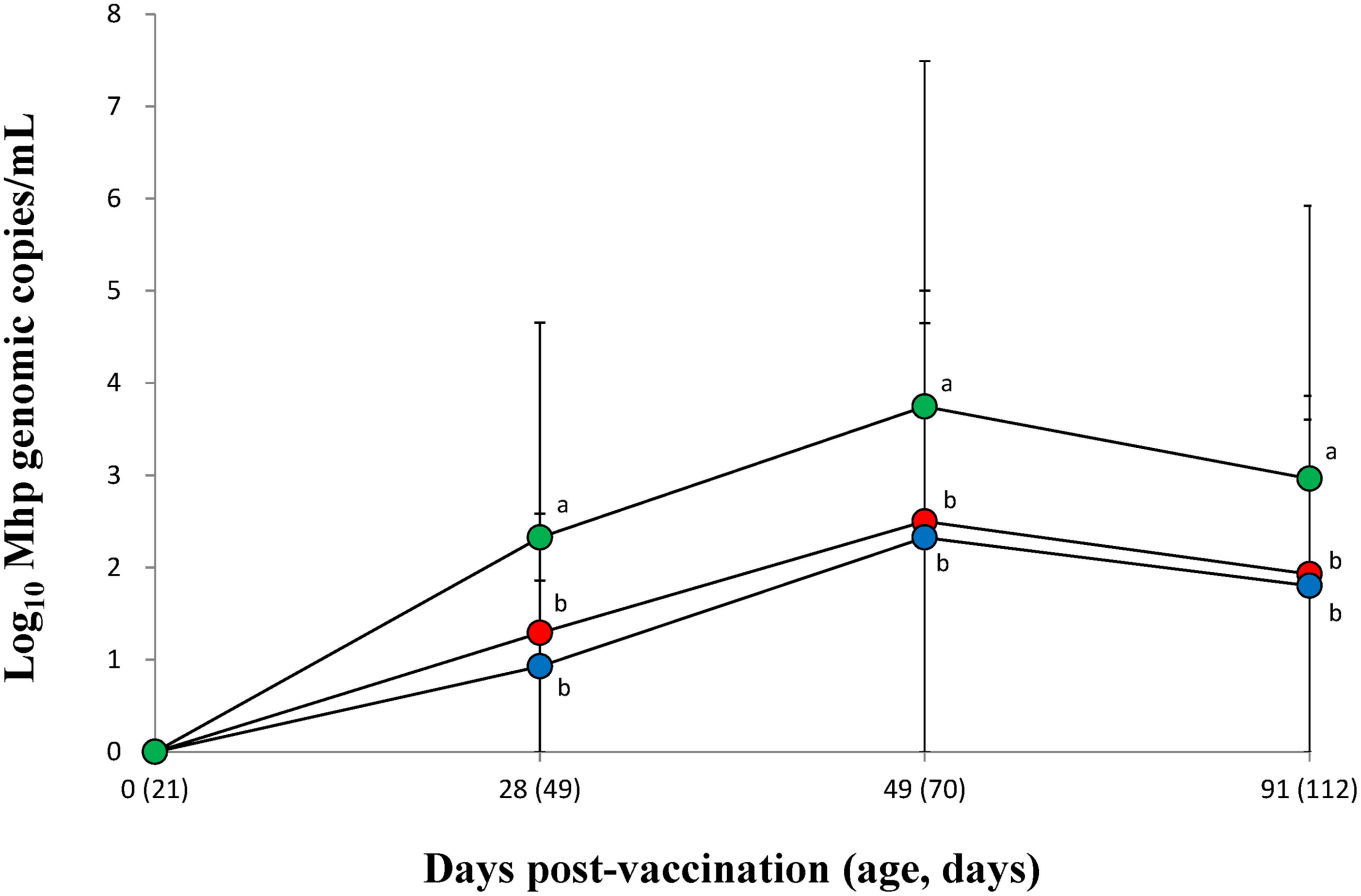
Mean values of the genomic copy number of *Mycoplasma hyopneumoniae* DNA in the larynx of pigs from the VacA (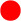), VacB (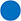), and UnVac (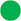) groups. Variation is expressed as the standard deviation (SD). Different superscripts (a and b) indicate a significant (*p* < 0.05) difference between vaccinated (VacA and VacB) and unvaccinated (UnVac) groups.

The amount of *M. hyopneumoniae* DNA loads in the larynx was significantly (*p* < 0.05) lower in the vaccinated groups (VacA and VacB) than in those of the unvaccinated group (UnVac) between 28 and 91 dpv (Figure 2). Throughout the entire field trial, two vaccinated groups (VacA and VacB) had comparable *M. hyopneumoniae* DNA loads in their larynx.

### Immune Responses Against PCV2

**Figure 3:**
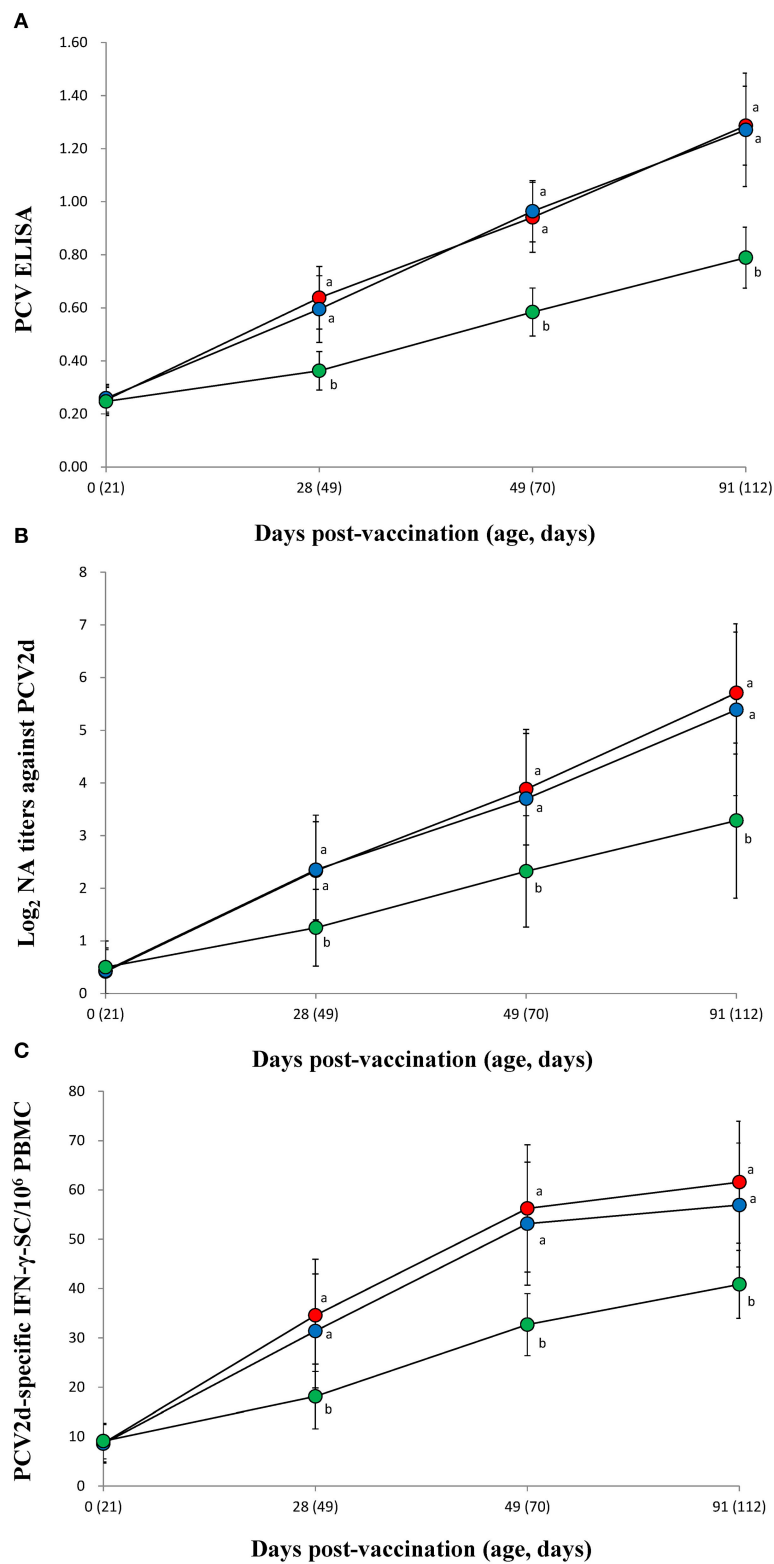
Immune responses against porcine circovirus type 2 (PCV2). **(A)** Mean values of the ELISA anti-PCV2 antibodies. **(B)** Mean values of the neutralizing antibody (NA) titers. **(C)** Frequency of PCV2d-specific interferon-γ secreting cells (IFN-γ-SC) from the VacA (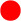), VacB (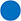), and UnVac (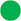) groups. Variation is expressed as the standard deviation (SD). Different superscripts (a and b) indicate a significant (*p* < 0.05) difference between vaccinated (VacA and VacB) and unvaccinated (UnVac) groups.

The vaccinated groups (VacA and VacB) were measured significantly (*p* < 0.05) higher in their PCV2 ELISA titers (Figure 3A), neutralizing antibody titers (Figure 3B), and IFN-γ-SC (Figure 3C) than that of the unvaccinated (UnVac) group at 28, 49, and 91 dpv. No significant differences in PCV2 ELISA titers, neutralizing antibody titers, or IFN-γ-SC were observed in the two vaccinated (VacA and VacB) groups throughout the entire field trial.

### Immune Responses Against *M. hyopneumoniae*

**Figure 4:**
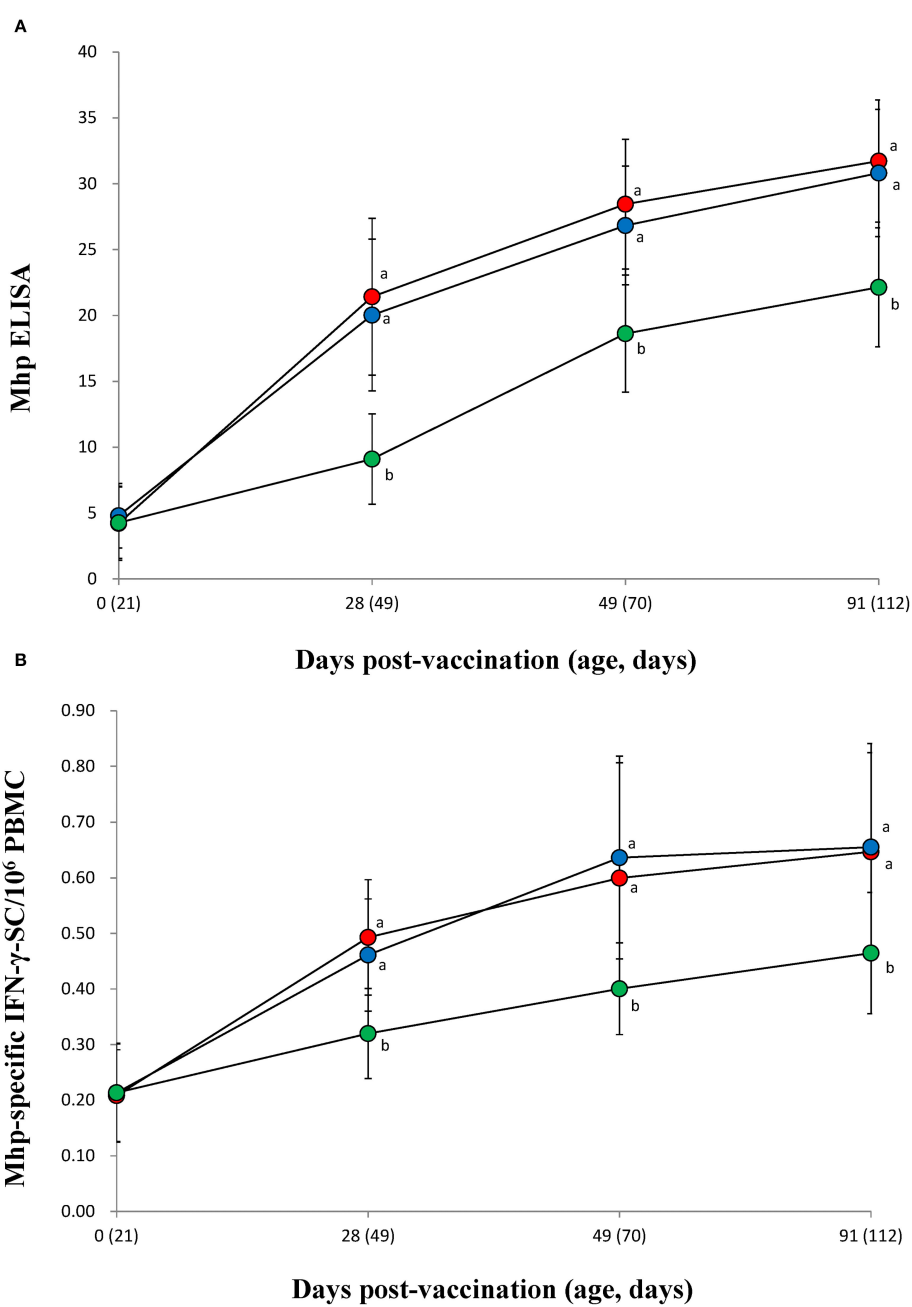
Immune responses against *Mycoplasma hyopneumoniae*. **(A)** Mean values of the anti-*M. hyopneumoniae* antibodies. **(B)** Frequency of *M. hyopneumoniae*-specific interferon-γ secreting cells (IFN-γ-SC) of pigs from the VacA (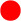), VacB (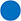), and UnVac (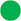) groups. Variation is expressed as the standard deviation (SD). Different superscripts (a and b) indicate a significant (*p* < 0.05) difference between vaccinated (VacA and VacB) and unvaccinated (UnVac) groups.

Both vaccinated groups (VacA and VacB) were measured significantly (*p* < 0.05) higher in their *M. hyopneumoniae* ELISA S/P ratios (Figure 4A) and IFN-γ-SC levels (Figure 4B) than animals from the UnVac group at 28, 49, and 91 dpv. No significant differences in *M. hyopneumoniae* ELISA S/P ratios or IFN-γ-SC were observed in the two vaccinated (VacA and VacB) groups throughout the entire field trial.

### Pathology

Vaccination of animals from both vaccinated groups (VacA and VacB) effectively reduced macroscopic lung lesion scores, microscopic lung, lymphoid lesion scores, and the numbers of lymphoid PCV2-positive cells when compared to the unvaccinated group (UnVac) at 154 dpv (Table 2). Throughout the entire field trial, there were no significant differences in overall scores for macroscopic and microscopic lung lesions, microscopic lymphoid lesions, and the numbers of lymphoid PCV2-positive cells between the two vaccinated groups (VacA and VacB).

**Table 2:** Growth performance with average daily weight gain (ADWG) and pathology between vaccinated and unvaccinated animals.

	**Age (days)**	**Days post vaccination**	**Groups**		
			**VacA**	**VacB**	**UnVac**
ADWG	21–70	0–49	403.03 ± 26.39	401.84 ± 22.33	387.48 ± 23.14
(gram/pig/day)	70–175	49–154	767.83 ± 17.99 ^a^	764.86 ± 17.58 ^a^	717.24 ± 17.02 ^b^
	21–175	0–154	651.79 ± 10.06 ^a^	649.33 ± 9.62 ^a^	612.29 ± 10.38 ^b^
Body weight	21	0	5.49 ± 0.35	5.49 ± 0.35	5.46 ± 0.32
	175	154	105.87 ± 1.50 ^a^	105.48 ± 1.40 ^a^	99.75 ± 1.59 ^b^
Macroscopic	175	154	16.91 ± 4.45 ^a^	17.96 ± 4.85 ^a^	29.20 ± 9.39 ^b^
lung lesions					
Microscopic	175	154	0.78 ± 0.50 ^a^	0.91 ± 0.56 ^a^	2.12 ± 0.65 ^b^
lung lesions					
Microscopic	175	154	0.74 ± 0.60	0.79 ± 0.71	1.08 ± 0.80
lymphoid lesions					

*Different superscripts (a and b) mean statistically significant differences within vaccinated and unvaccinated groups (p < 0.05)*.

## Discussion

The results of this comparative field trial demonstrate that PCV2a- and PCV2b-based bivalent vaccines that also contain *M. hyopneumoniae* provide equal protection for pigs in herds suffering from subclinical PCV2d infection and enzootic pneumonia. The common denominator of PCV2d and *M. hyopneumoniae* infection is weight loss, so it was important to evaluate the improvement of growth performance in the comparative field trial. Vaccination of pigs with both evaluated bivalent vaccines (two groups) resulted in a significantly improved growth performance as compared to the pigs in the unvaccinated group. Significant differences in growth performance as measured by body weight and ADWG were not found between the two bivalent-vaccinated groups.

In general, both PCV2/*M. hyopneumoniae* combination vaccines induced protective immunity by reducing PCV2 blood viral load and *M. hyopneumoniae* laryngeal load while simultaneously reducing lung and lymphoid lesions, thereby controlling these two diseases (32–34). A PCV2b-based bivalent vaccine may provide better protection in theory against PCV2d than PCV2a-based bivalent vaccines as PCV2b is closely related to PCV2d genetically (10, 20). In the present study, both bivalent vaccines elicited equal levels of neutralizing antibodies and IFN-γ-SC against PCV2d while simultaneously reducing the level of PCV2d blood viral load. Genetic similarity therefore does not guarantee that one bivalent vaccine can offer superior protection over the other. Like the PCV2 response with vaccination, both bivalent vaccines elicited an equal level of IFN-γ-SC against *M. hyopneumoniae* while simultaneously equally reducing the levels of *M. hyopneumoniae* laryngeal load. Besides antigen genotype, several other factors, such as adjuvant, vaccine formulation, and route of administration, also influence the immune responses and efficacy of a vaccine.

The immunological and microbiological findings in this study were consistent with the pathological findings. The pathological analysis is critical in the evaluation and comparison of bivalent vaccine efficacy because pathological lesion severity is the critical criterion for the diagnosis of PCVAD. The reduction in lung and lymphoid lesions due to *M. hyopneumoniae* and PCV2d infection is correlated with growth performance (35–38). Both bivalent vaccines reduced lung and lymphoid lesions without any clear advantages of one vaccine over the other. Therefore, it was confirmed that both vaccines efficiently reduced lung lesions caused by *M. hyopneumoniae* and lymphoid lesions caused by PCV2d (35–38).

Porcine circovirus type 2 is a virus that has the highest mutation rate among all DNA viruses (39). The high mutation rate of PCV2 results in the emergence of new genotypes. Currently, PCV2d is the most prevalent genotype found in all major pigs rearing Asian countries (13–16). To date, a commercial bivalent vaccine containing PCV2d and *M. hyopneumoniae* is not yet available. Alternatively, it is necessary to compare the differences in clinical, immunological, microbiological, and pathological results between PCV2a- and PCV2b-based bivalent vaccines and how they result in farm outbreaks with subclinical PCV2d infection and enzootic pneumonia. These comparative field data indicate that both bivalent vaccines are good candidates for controlling disease in swine farms suffering from subclinical PCV2d infection and enzootic pneumonia.

## Data Availability Statement

The datasets presented in this study can be found in online repositories. The names of the repository/repositories and accession number(s) can be found in the article/supplementary material.

## Ethics Statement

The animal study was reviewed and approved by Seoul National University Institutional Animal Care and Use Committee (SNU-210518-3).

## Author Contributions

HC and TO contributed to the performance of the experimental trials, data analysis, and writing of the manuscript. HC, TO, and JS contributed to the preparation of the inoculum and lab analysis. CC contributed to the development of the protocol, design of the study, review of the final manuscript, and the approval for publication. All authors read and approved the final manuscript.

## Funding

The author's research was supported by contract research funds (Grant No. 550-20190068) of the Research Institute for Veterinary Science (RIVS) from the College of Veterinary Medicine and by the BK 21 Plus Program (Grant No. 5260-20150100) for Creative Veterinary Science Research.

## Conflict of Interest

The authors declare that the research was conducted in the absence of any commercial or financial relationships that could be construed as a potential conflict of interest.

## Publisher's Note

All claims expressed in this article are solely those of the authors and do not necessarily represent those of their affiliated organizations, or those of the publisher, the editors and the reviewers. Any product that may be evaluated in this article, or claim that may be made by its manufacturer, is not guaranteed or endorsed by the publisher.
